# Oscillation theorems for second order nonlinear forced differential equations

**DOI:** 10.1186/2193-1801-3-300

**Published:** 2014-06-18

**Authors:** Ambarka A Salhin, Ummul Khair Salma Din, Rokiah Rozita Ahmad, Mohd Salmi Md Noorani

**Affiliations:** School of Mathematical Sciences, Faculty of Science and Technology, Universiti Kebangsaan Malaysia, 43600 UKM Bangi, Selangor, Malaysia

**Keywords:** Oscillation, Forced nonlinear differential equations of second order

## Abstract

In this paper, a class of second order forced nonlinear differential equation is considered and several new oscillation theorems are obtained. Our results generalize and improve those known ones in the literature.

## Introduction

We consider the oscillation behavior of solutions of second order forced nonlinear differential equation
1.1

and
1.2

where *r*, *q* ∈ *C*([*t*_0_, ∞), *ℝ*), and *f*, *ψ*, *g* ∈ *C*(*ℝ*, *ℝ*) and *H* is a continuous function on [*t*_0_, ∞) × *ℝ*^2^,

*α* is a positive real number. Throughout the paper, it is assumed that the following conditions are satisfied:

(A_1_) *r*(*t*) > 0, *t* ≥ 0;

(A_2_) *xg*(*x*) > 0, *g* ∈ *C*^1^(*ℝ*) *for x* ≠ 0;

(A_3_) 

We restrict our attention only to the solutions of the differential equations (1.1) and (1.2) that exist on some ray [*t*_0_, ∞), where *t*_0_ ≥ *t*, to may depend on the particular solutions. Such a solution is said to be oscillatory if it has arbitrarily large zeros, and otherwise, it is said to be nonoscillatory. Equations (1.1) and (1.2) are called oscillatory if all its solutions are oscillatory.

The problem of finding oscillation criteria for second order nonlinear ordinary differential equations, which involve the average of integral of the alternating coefficient, has received the attention of many authors because in the fact there are many physical systems are modeled by second order nonlinear ordinary differential equations; for example, the so called Emden – Fowler equation arises in the study of gas dynamics and fluid mechanics. This equation appears also in the study of relativistic mechanics, nuclear physics and in the study of chemically reacting systems.

The oscillatory theory as a part of the qualitative theory of differential equations has been developed rapidly in the last decades, and there has been a great deal of work on the oscillatory behavior of differential equations; see e.g. (Agarwal et al. 2010; Beqiri and Koci 2012; Bihari 1963; Elabbasy and Elsharabasy 1997; Elabbasy and Elhaddad 2007; Grace et al. 1984, 1988; Grace and Lalli 1987, 1989, 1990; Grace 1989, 1990, 1992; Greaf and Spikes 1986; Graef et al. 1978; Lee and Yeh 2007; Kamenev 1978; Kartsatos 1968; Li and Agarwal 2000; Meng 1996; Nagabuchi and Yamamoto 1988; Ohriska and Zulova 2004; Ouyang et al. 2009; Philos 1983, 1984, 1985; Remili 2010; Salhin 2014; Tiryaki and Basci 2008; Tiryaki 2009; Temtek and Tiryaki 2013; Yan 1986; Yibing et al. 2013a, [b]; Zhang and Wang 2010).

Remili (2010), studied the equation
1.3

and derived some oscillation criteria for the equation (1.3), where new results with additional suitable weighted function are investigated. Zhang and Wang (2010), studied the following equation
1.4

Temtek and Tiryaki (2013) obtained several new oscillation results for the equation
1.5

and its special cases by using generalized Riccati transformation and well known techniques.

In this paper, we continue in this direction the study of oscillatory properties of equations (1.1) and (1.2). The purpose of this paper is to improve and extend the above mentioned results. Our results are more general than the previous results. The relevance of our results becomes clear due to some carefully selected examples.

## Main results

In this section we prove our main results.

**Theorem 2.1.** Suppose that, conditions (A_1_) – (A_3_) hold, and
2.12.2

Let *ρ* be a positive continuously differentiable function over [*T*, ∞) such that *ρ*^′^(*t*) ≥ 0 over [*T*_0_, ∞); 
2.32.4

where

Then all solutions of equation (1.1) are oscillatory.

**Proof.** Let *x*(*t*) be a non-oscillatory solution on [*T*, ∞), *T* ≥ *T*_0_ of the equation (1.1). We assume that *x*(*t*) is positive on [*T*, ∞), *T* ≥ *t*_0_. A similar argument holds for the case when *x*(*t*) is negative. Let
2.5

Then differentiating (2.5), (1.1) and take in account assumptions (A_1_) - (A_3_), (2.2) we have
2.6

In view of (2.1) we conclude that
2.7

By using the extremum of one variable function it can be proved that


Now, by applying this inequality we have
2.8

Integrating (2.8) from *T* to *t*, we get
2.9

Taking the limit for both sides of (2.9) and using (2.4), we find *w*(*t*) → − ∞. Hence, there exists *T*_1_ ≥ *T* such that *f*(*x*^′^(*t*)) < 0 ⇒ *x*^′^(*t*) < 0, ∀*t* ≥ *T*_1_.

Condition (2.4) also implies that  and there exists *T*_2_ ≥ *T*_1_such that

 and 

Multiplying Eq. (1.1) by *ρ*(*t*) and integrating by parts on [*T*_2_, *t*], we have


Now, integrating by parts, we get


where


Therefore,


From (2.1) and (2.2), we find


From (2.3) and 0 < *x*(*t*) ≤ *x*(*T*_2_), this implies that  is lower bounded, but the right side of it tends to mines infinity. Then, this is a contradiction.

**Example 2.2.** Consider the following differential equation


Evidently, if we take  Then all conditions of Theorem 2.1 are satisfied, hence, all the solutions are oscillatory.

**Theorem 2.3.** If (A_1_) – (A_3_), conditions (2.1) – (2.3) hold, and
2.102.112.12

and
2.13

Thus all solutions of Eq. (1.1) are oscillatory.

**Proof.** Let *x*(*t*) be a non-oscillatory solution on [*T*, ∞), *T* ≥ *T*_0_ of Eq. (1.1). Let us assume that *x*(*t*) is positive on [*T*, ∞) and consider the following three cases for the behavior of *x*^′^(*t*).

Case 1: *x*^′^(*t*) > 0 for *T*_1_ ≥ *T* for some *t* ≥ *T*_1_; then from (2.10), we obtain


From (2.1) and (2.2), we obtain


Hence, for all *t* ≥ *T*_1_

Using (2.13), we obtain


which contradicts to the condition (2.13).

Case 2: If *x*^′^(*t*) is oscillatory, then there exists a sequence {*α*_*n*_} → ∞ on [*T*, ∞) such that *x*^′^(*α*_*n*_) < 0. Let us assume that *N* is sufficiently large so that


Then, from (2.1), (2.2) and (2.7), we have


Thus


which contradicts to the assume that *x*^′^(*t*) oscillates.

Case 3: Let *x*^′^(*t*) < 0 for *t* ≥ *T*_1_. Condition (2.11) implies that for any *t*_0_ ≥ *T*_1_ such that

 for all *t* ≥ *T*_1_.

The remaining part of the proof is similar to that of Theorem 2.1 then will be omitted.

**Example 2.4.** Let us consider the following equation


Evidently, if we take  Then the equation given in Example 2.2 is oscillatory by Theorem 2.2.

**Remark 2.1.** Condition (2.10) implies that  and  hence (2.11) takes the form of  for all large *T*.

**Remark 2.2.** when *α* = 1, *ψ*(*x*(*t*)) = 1 and *f*(*x*′(*t*)) = *x*′(*t*), Theorem 2.1 and 2.2 reduce to Theorem 1 and 2 Remili (2010) and Theorems 2.1 and 2.3 are obtained by analogy with Theorems 2.1 and 2.2 from (Temtek and Tiryaki 2013).

**Theorem 2.5.** Assume that
2.142.15

Furthermore, assume that there exist a constant *A* such that
2.16

where 2.17

Then the differentia Eq. (1.2) is oscillatory.

**Proof.** Without loss of generality, let assume that there exists a solution *x*(*t*) of (1.2) such that *x*(*t*) > 0 on [*T*, ∞) for some *T* ≥ *t*_0_. A similar argument holds also for the case when *x*(*t*) < 0. Let *w*(*t*) be defined by the Riccati Transformation


Derivation this equality we have


This, and (1.2) imply


Integrating this inequality from *T* to *t*( ≥ *T*), we obtain


By condition (2.14), we get


Integrating the above inequality multiplied by  from *T* to *t*( ≥ *T*), we have


From condition (2.16) and (2.17), we get that


Now, if *x*(*t*) ≥ *x*(*T*) for large *t* then *θ*(*t*) ≥ 0, which is a contradiction. Hence for large *t*, *x*(*t*) ≤ *x*(*T*), so


which is again a contradiction. This completes proof the Theorem 2.3.

**Theorems 2.6.** Suppose that conditions (2.14), (2.15) and (2.16) hold. Furthermore, suppose that, there exist a function *ρ* : [*t*_0_, ∞) → (0, ∞) such that *ρ*′(t) ≥ 0 for all t ≥ t_0_, and
2.18

Then the differential equation (1.2) is oscillatory.

**Proof.** Without loss of generality, let assume that there exists a solution *x*(*t*) of (1.2) such that *x*(*t*) > 0 on [*T*, ∞) for some *T* ≥ *t*_0_. Let *w*(*t*) be defined by the Riccati Transformation


Derivation this equality we have


This, and (1.2) imply


Hence for all *t* ≥ *T*, we obtain
2.19

By the Bonnet’s Theorem that for each *t* ≥ *T*, there exist a *T*_0_ ∈ [*T*, *t*] such that
2.20

By (2.19) and (2.20) we get
2.21

Integrating the above inequality multiplied by  from *T* to *t*( ≥ *T*), we obtain


From (2.16) and (2.18), we have


Now, if *x*(*t*) ≥ *x*(*T*) for large *t*, then *θ*(*t*) ≥ 0, which is a contradiction. Hence for large *t*, *x*(*t*) ≤ *x*(*T*), so


which is again a contradiction. This completes proof the Theorem 2.6.

**Example 2.7.** Consider the differential equation


Here,


So, can note that


Let us take *ρ*(*t*) = 1 we have


then, Theorem 2.4 ensures that every solution of the equation given oscillates.
